# Physicochemical, Sensory, and Cooking Qualities of Pasta Enriched with Oat β-Glucans, Xanthan Gum, and Vital Gluten

**DOI:** 10.3390/foods9101412

**Published:** 2020-10-05

**Authors:** Ada Krawęcka, Aldona Sobota, Emilia Sykut-Domańska

**Affiliations:** Department of Plant Food Technology and Gastronomy, Division of Engineering and Cereals Technology, University of Life Sciences, Skromna 8 Street, 20-704 Lublin, Poland; ada.krawecka@gmail.com (A.K.); emilia.sykut-domanska@up.lublin.pl (E.S.-D.)

**Keywords:** functional food, fortified pasta, β-glucans, nutritional properties, sensory analysis

## Abstract

The functional properties of β-glucans derived from oats and barley are confirmed by numerous in vitro and in vivo studies. This study aimed to assess the effect of adding 0, 5, 10, 15, and 20% oat (1,3)(1,4)-β-D-glucans to physicochemical properties, as well as the cooking and sensory qualities of durum wheat pasta. Additionally, to improve the cooking and sensory qualities of pasta, we added 5% of xanthan gum and vital gluten. The present study showed that the addition of β-glucans led to an increase of the water absorption index (WAI), water solubility index (WSI), and viscosity of products. At the same time, an increase in the content of fat, ash, and dietary fiber was observed. The addition of (1,3)(1,4)-β-D-glucans influenced the cooking quality of the pasta, extending the minimum cooking time and increasing the loss of dry matter. At the same time, the color of the product changed. In the case of cooked pasta, the addition of β-glucans decreased the brightness and increased the yellowness and redness. It was found that the products enriched with 10–15% of β-glucans, as well as 5% of xanthan gum and vital gluten would yield functional pasta that may offer health benefits beyond its nutritional value. Further, this could influence high cooking and sensory quality.

## 1. Introduction

Functional food is part of the fastest growing sector of the global food market and is a response to the growing consumer demand for health products [1]. According to the European Commission, the term “functional food” denotes food that not only has nutritional effects but also exerts a beneficial effect on the physical functions of the body and, in some cases, reduces the risk of specific diseases. These beneficial effects must be confirmed by scientific research. Functional food must have a form easily accessible to the consumer in order to be part of the daily diet [2]. On an industrial scale, these are usually products to which a health-promoting component has been added, its bioavailability has been increased, or an adverse component has been removed [3,4,5].

A poorly balanced diet has a great impact on the development of chronic non-communicable diseases (NCD). There is still an upward trend in the incidence of type 2 diabetes and cardiovascular diseases [6,7]. An integral part of preventing these diseases is to increase dietary fiber intake [8,9,10]. Several studies have emphasized that high consumption of cereal-derived fiber is associated with a reduction in the risk of development of type 2 diabetes [11,12,13]. Dietary fiber has been used for fortification for many years. Its soluble fractions (SDF) are thought to be extremely functional. They increase viscosity in the stomach and delay its emptying. In the intestines, they create a barrier to enzymes and consistently slow down the hydrolysis of nutrients and absorption of glucose and cholesterol from food [14,15,16,17]. What is more, soluble fiber is easily fermented by bacteria living in the colon, resulting in the production of short-chain fatty acids (SCFAs), which lower the pH of the environment and stimulate the development of beneficial microflora [13]. The soluble fiber fraction includes (1,3)(1,4)-β-D-glucans, which are polymers of glucose present in the cell walls of cereal grains, especially barley and oats [18]. These ingredients have been documented to exert pro-health effects and may be used as a functional component in food [19]. As reported by Jenkins et al. [20], a 1-g increase in the content of β-glucans in a product reduces a food’s glycemic index (GI) by 4 units. A number of scientific studies confirmed that a 4–6-week diet based on products with a low glycemic index significantly reduces the fasting blood glucose level and insulin secretion, in addition to increasing insulin sensitivity. Concurrently, it reduces the level of glycated hemoglobin (HbA1c); hence, it is an effective method to prevent and treat diabetes [21,22,23]. The consumption of β-D-glucans at the level of 4 g/30 g of digestible carbohydrates present in a meal helps reduce postprandial glucose, while the consumption at a level of 3 g/day helps maintain normal blood cholesterol levels [24].

Pasta produced from semolina durum or common wheat flour is one of the most popular cereal products and can be a suitable food matrix for fortification with functional ingredients. Many studies focus on the possibility of enriching pasta with high-fiber raw materials, including oat flour, β-glucan concentrates [25,26,27,28,29,30,31], legume components [32], or pomaces [33,34]. It should be noted that the addition of both insoluble and soluble fractions of dietary fiber can weaken the protein-starch matrix and has a negative effect on the cooking and textural qualities of pasta [17,34,35]. However, some high-fiber materials such as xanthan gum or high-protein material (e.g., vital gluten) can improve dough strength and the cooking and sensory qualities of pasta [36,37,38,39,40,41]. At the same time, these components may improve the health-promoting value of a product. The effects of adding β-glucans to pasta have been examined by other authors, but there is no research on the possibility of reducing the negative effect of this component addition on pasta sensory and cooking qualities. For this reason, the aim of the study was to determine the possibility of using oat β-glucans and additionally xanthan gum and vital gluten to obtain functional pasta with high quality properties and health benefits.

## 2. Material and Methods

### 2.1. Characteristics of Raw Materials

The raw material used in the study was semolina durum (Julia Malom, Kunszállás, Hungary), from which the control sample was produced (i.e., pasta without additives (CON)). Subsequent samples were supplemented with the same level of xanthan gum (5%) (Agnex, Białystok, Poland) and vital wheat gluten (5%) (Polmarkus, Pyskowice, Poland) to the semolina. The oat β-glucans supplement (Brenntag Polska Sp.z o.o., Kędzierzyn-Koźle, Poland) was variable and amounted to 0, 5, 10, 15, and 20% (samples BG0, BG5, BG10, BG15, BG20, respectively). Samples of raw materials weighing 5 kg were moistened to 33% humidity. The detailed model of the experiment is presented in Table 1.

### 2.2. Pasta Preparation

Fusilli pasta was produced in a semi-technical laboratory scale using a MAC-30S Lab pasta extruder (ItalPast, Parma, Italy). The ingredients of the dough were premixed for 15 min under atmospheric pressure and, subsequently, the dough was mixed and extruded under a vacuum (0.086 MPa). The rotational speed of the screw of the pasta extruder was 48 rpm. The pasta samples were dried at a controlled temperature and humidity in a pasta dryer EAC30-LAB (ItalPast, Parma, Italy) in conditions described previously by Sobota et al. [42].

### 2.3. Chemical Analysis

The analysis of the chemical composition was performed using American Association of Cereal Chemists Approved Methods (AACC) and Association of Official Analytical Chemists International (AOAC) methods [43,44]. The moisture content was determined with the air-oven method (Method AACC 44-15A). The samples (3 g) were placed in a laboratory dryer and dried at 103 °C ± 1° C to constant weight. After cooling in an exsiccator, the samples were weighed and the moisture contents were calculated. The ash content was determined using AACC method 08–01. The samples were measured into ash dishes at 3 g. Then, the samples were placed in a muffle furnace at 550 °C. They were incinerated until light gray ash or constant weight was obtained (7 h). After cooling, the samples were weighed and the ash contents were calculated. To determine the total protein content, the Kjeldahl method (Method AACC 46-08) and the Kjeltec 2300 (FOSS, Höganäs, Sweden) apparatus were used. The protein content was calculated from the total nitrogen content using converted factor 5.7. The crude fat content was determined via continuous extraction. The SoxtecTM8000 on application AN 310 (FOSS, Höganäs, Sweden) and hexane as a solvent were used. The total dietary fiber (TDF) content, including insoluble dietary fiber (IDF) and soluble dietary fiber (SDF), according to the enzymatic methods (AACC 32-05, AACC 32-21, AOAC 991.43, and AOAC 985.29) was analyzed. Next, 1 g dried samples were subjected to sequential enzymatic digestion using heat-stable α-amylase, protease, and amyloglucosidase. Megazyme enzymes and analytical procedures were used (Megazyme International Ireland Ltd., Wicklow, Ireland). The digestible carbohydrate content was determined by calculating the difference (weight in grams (protein + fat + TDF + ash) in 100 g of dry matter of pasta or raw material).

### 2.4. Physical Properties

The quality parameters of semolina (i.e., wet gluten content, gluten elasticity, and gluten spreadability) were tested in accordance with the Polish Standard (PN-92/A-74021) [45]. The granulometric composition of durum semolina was determined with the sieve method using a laboratory sieve shaker (Sadkiewicz Instruments—Bydgoszcz) and a set of sieves with the following mesh sizes: 400, 315, 250, 160, 125, and 80 µm. Next, 100 g of samples was sieved for 10 min. The percentage share of each fraction in the semolina and the equivalent diameter (weighted average grain diameter in the sample) were calculated. The details of the method were described by Sobota et al. [35]. In the case of fine-grained raw materials (i.e., vital wheat gluten, xanthan gum, and oat β-glucans), the particle size was examined with microscopic image analysis using an optical microscope (×40 magnification) and DLT CAMViewer 3.7.4043 software. Raw material samples were prepared and the diameters of 70 randomly selected raw material particles were measured, and their average diameter was calculated. The water solubility index (WSI) and the water absorption index (WAI) were investigated according to the centrifuge method (AACC 56-20), with slight modification described by Sobota et al. [35]. Further, 2 g of samples were placed in centrifuge tubes and mixed with 30 mL of distilled water. The suspension was left to rest for 5 min then was centrifuged for 15 min at 2200× *g*. Next, 10 mL of supernatant was dried to the solid mass and the WSI was calculated using the Formula (1):

(1) $$\mathrm{WSI}\;(\%)\;=\;(\mathrm{Weight}\;\mathrm{of}\;\mathrm{dried}\;\mathrm{supernatant}\;\times \;\frac{30\;\mathrm{mL}}{10\;\mathrm{mL}}/\mathrm{Dry}\;\mathrm{weight}\;\mathrm{of}\;\mathrm{sample})\;\times \;100\%.$$

After 10 mL of the supernatant was downloaded to determine the WSI, the remaining supernatant was carefully decanted. The wet samples were weighed and WAI was calculated using the Formula (2):

WAI (%) = ((Weight of wet sample − Weight of dry sample)/Dry weight of sample) × 100%.(2)

Apparent viscosity was tested in accordance with the method developed by Zarzycki and Sobota [46]. A rotary rheometer RM 180 (Mettler-Toledo AG, Switzerland, software RSI Orchestrator, ver. V6.5.8.) with coaxial cylinders without the bottom cylinder guard (shear rate of 1000 s^−1^) was used. A 5% suspension in distilled water at 30 °C was prepared from ground pasta samples. The suspensions were held for 30 min at 30 °C with constant stirring. The suspensions (300 mL) were heated in a laboratory shaker (Elpin type 357, Elpin Plus s.c., Lubawa, Poland). The viscosity was measured at a temperature ranging from 65 to 95 °C; next, they were kept at 95 °C for 20 min, cooled to 50 °C, and kept for 30 min at this temperature. A constant temperature gradient of 1 °C min^−1^ was maintained during the heating and cooling processes. When the viscosity measuring agitation was stopped, the cylinders were immersed in the suspension, and five consecutive readings were taken every 10 s. The measurements were made in 3 replications for each sample.

### 2.5. Cooking Quality of Pasta Samples

The optimal cooking time (OCT, min) was measured according to Method AACC 16–50 [43]. Next, 50 g of pasta was boiled in 500 mL of distilled water. Every 30 s, the pasta was removed and squeezed between two glass plates until the mealy core disappeared. The time needed for this process was assumed as the optimal cooking time (OCT). The weight increase index (WI) was calculated by dividing the weight of the pasta sample after cooking by the weight of the uncooked pasta sample (50 g) [47]. In order to determine the volume increase index (VII), the volume of the pasta was tested by dipping a 50 g sample of an uncooked product in a measuring cylinder filled with 400 mL of vegetable oil. The volume increase was equal to the volume of the tested pasta sample. A sample of pasta (50 g) was then cooked and the volume of the cooked product was determined in an analogous manner. VII was calculated by dividing the volume of cooked pasta by the volume of an uncooked product. Cooking loss (CL, g/100 g d.m.) was determined by testing the dry matter content in water after cooking a 50 g pasta sample. The dry matter content in water was determined according to the AACC 44-15A method [43].

### 2.6. Color of Pasta

The color of the cooked and uncooked pasta samples was measured using a colorimeter (X-Rite 8200, Inc., Grand Rapids, MI, USA) with a standard light source (D65), a standard colorimetric observer (10°), and a 12.3 mm diameter hole. White and black calibration references were applied to standardize the instrument before analysis. The following CIE parameters were recorded: L* (lightness, indicates the level of light 100 or dark 0), a* (−a* = indicates greenness, +a* = indicates redness), and b* values (−b* = blue, +b* = yellow). The measurements were performed repeatedly 10 times per each sample.

### 2.7. Sensory Analysis

The sensory analysis was carried out in accordance with the method described by Sozer et al. [48]. The analysis involved 12 people (8-females and 4-males, 23–48 years old), who had adequate taste sensitivity. The panelists had been previously trained how to evaluate the sensory parameters of pasta: appearance (regularity of shape, lack of deformation, cracks and scratches), color (should be regular and light-yellow), odor and taste (should be characteristic and similar to that of durum semolina pasta), hardness (evaluated as a resistance of cooked pasta to compression by the teeth), adhesiveness (evaluated by placing in the mouth, pressing it against the palate, and determining the force required to remove it with the tongue), springiness (was measured as the degree to which the product returns to its original shape after partial compression). Properly coded samples were cooked for OCT in random order and evaluated within a time no longer than 5 min after cooking.

A 5-point rating scale was used, in which 5 was the maximum score. Assuming that all evaluated parameters had equal weight, the average sensory rating was calculated for each pasta sample.

### 2.8. Statistical Analysis

The obtained results were subjected to statistical analysis using the statistical program STATISTICA 13.1 (StatSoft ©, Inc. Tulsa, USA). All experimental results were means (± S.D) from at least three assays. One-way analysis of variance (ANOVA) and Tukey’s post-hoc test were used to compare the groups. The results were statistically different for *p*-values < 0.05.

## 3. Results and Discussion

### 3.1. Pasta Processing

**Figure 1:**
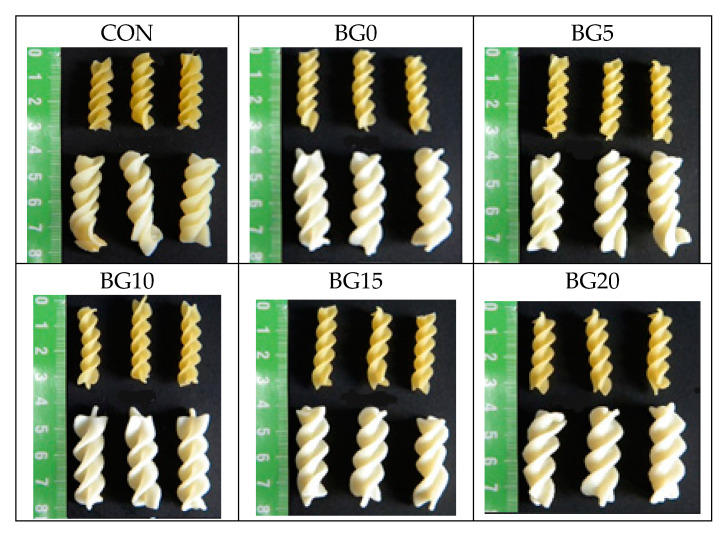
Uncooked and cooked pasta samples. CON—control sample; BG—β-glucans.

The addition of β-glucans, vital gluten, and xanthan gum affected the extrusion process of the pasta. The increasing content of β-glucans in the enriched pasta resulted in higher pressure values, compared to the control sample (Table 1). The dough was pushed through a dye under diminished pressure from 8.5 MPa for the control pasta to 13 MPa (BG0, BG5, BG10) and 13.5 MPa (BG15, BG20). The addition of gluten and xanthan gum caused a significant reduction in the efficiency of the pasta extruder. The formation of a strong gluten matrix caused by the addition of vital gluten and xanthan gum limited the flow rate of the dough through the forming holes in the die and thus reduced the extruder’s efficiency. Additionally, the high water absorption of xanthan gum and gluten changed the consistency of the dough. It became harder and less plastic. The increase in the β-glucan content increased the plasticity of the dough. The relatively high content of soluble fiber and fat in this raw material made it difficult to build a strong protein-starch matrix in the dough. The flow rate of the dough through the holes in the matrix increased and, consequently, the extruder’s performance increased. Pasta samples (uncooked and cooked) obtained in this study are shown in Figure 1.

### 3.2. Chemical Analysis

The chemical composition of the raw materials and pasta samples is reported in Table 2. The high content of protein in semolina was responsible for the creation of the strong protein-starch matrix in pasta, which determined the cooking and product quality parameters [22]. In the case of pasta fortified with high-fiber raw materials, weakening of the gluten network and deterioration of cooking quality parameters were most often noted [17,49]. Therefore, vital wheat gluten was used in the β-glucan-fortified pasta samples. The addition of vital wheat gluten, which contained up to 71% protein, caused an increase in the protein content in the sample BG0 and in all samples with β-glucans, compared to the control sample. The addition of β-glucans significantly (*p* ≤ 0.05) affected the chemical composition of the pasta. This raw material contained less protein than durum semolina; therefore, increasing the share of β-glucans from 0 to 20% at the expense of semolina led to a decrease in protein content. In addition, xanthan gum was added, which next to β-glucans was a good source of soluble dietary fiber [50]. At the same time, many studies have shown that a small addition of this component had a positive effect on the texture and sensory quality of cooked pasta [36,37,38,40,41]. As demonstrated by results presented by other authors, the addition of soluble dietary fiber (e.g., xanthan gum, guar gum) meant that, after hydration, non-starch polysaccharides surrounded the protein-starch network, reducing the loss of dry matter [38]. Along with the increase in β-glucans, the ash and fat content increased proportionally. The increase in the ash content resulted from the inclusion of the high-fiber raw materials (e.g., xanthan gum, β-glucans) in the product. The fat content was already high in the β-glucan preparation (Table 2). Both lipids and minerals had a positive effect on the cooking quality of pasta, increasing the stability of the starch-protein matrix [27]. Along with the increase in the share of β-glucans in the pasta, a significant (*p* ≤ 0.05) increase in the content of total fiber (TDF) and its soluble fraction (SDF) was noted. The values of TDF and SDF in the BG20 sample were more than four times and six times higher than the control sample, respectively. It should be emphasized that the addition of xanthan gum and gluten also caused a significant (*p* ≤ 0.05) increase in dietary fiber content in pasta. Enrichment of the product with β-glucans reinforced these trends. The addition of β-glucans caused a decrease in the share of digestible carbohydrates, which combined with a simultaneous increase in the content of the soluble fiber fraction, which could probably have an impact on reducing the glycemic index of pasta. Soluble fiber (β-glucans) competed with starch granules for water availability and reduced swelling and gelation of starch. As a result, its digestibility and availability decreased [28]. The glycemic index of the product was mainly influenced by the soluble fraction of dietary fiber. It increased the viscosity of the chyme, hindered the access of amylolytic enzymes to starch, reduced the dynamics of starch digestion, and created a sticky film on the intestinal surface impeding glucose absorption into the bloodstream. What is more, fiber stimulated the production of short-chain fatty acids (SCFA) that formed as a result of bacterial fermentation in the large intestine. These acids absorbed by colonocytes through the portal vein entered the liver, where they regulated the metabolism of fatty acids and cholesterol. Propionic acid was of particular importance, as it inhibited the synthesis of fatty acids that reached the bloodstream and regulated adipocytokines (adipokines), i.e., proteins responsible for *inter alia*, glycemic homeostasis, and lipidemia [10].

### 3.3. Physical Properties

The wet gluten content in the semolina was determined at 28.5%. The spreadability of gluten was 8 mm and the elasticity was estimated at I°. Strong gluten in durum wheat pasta determined its high cooking quality [51]. According to the quality requirements presented in the Polish Standard for Durum Wheat milling products, the minimum content of gluten in semolina durum should be 30% [45]. The raw material did not meet this requirement. Additionally, bearing in mind that the addition of high-fiber raw materials may additionally weaken the gluten network, the addition of vital gluten was used in the mixtures. The particle size of the individual raw materials varied within a wide range, which meant that their mean diameters did not differ significantly, except for the durum semolina (Table 3). The addition of β-glucans affected the water absorption index (WAI), water solubility index (WSI), and viscosity of pasta products (Table 3). The WAI of pasta with added β-glucans was over three times higher, compared to the control sample (CON); however, there were no significant (*p* ≤ 0.05) differences between the samples with β-glucans and the BG0 sample. 

The high content of dietary fiber (xanthan gum, β-glucans) present in these samples (Table 2) was associated with greater water absorption. As shown in literature reports, distortion of the protein network by the addition of dietary fiber can induce increased water absorption [26]. Foschia et al. [26] observed an increase in water absorption in pasta products when trying to replace semolina with dietary fiber raw materials. High-WAI products quickly satisfy hunger and maintain longer satiety [52]. In the present research, relatively low WSI values were observed for the products enriched with β-glucans. The values obtained may indicate a favorable low level of complex carbohydrate degradation during the production process. This assumption seems to be confirmed by the significantly (*p* ≤ 0.05) lower dynamics of digestible carbohydrates in the β-glucan enriched pasta samples (Table 2). Brennan et al. [53] found a positive correlation between WSI values of extruded cereal products enriched with high fiber fungal powder and glycemic response (glycemic index values). Low-WSI cereal products should not generate as high glycemic response as partially degraded starch polymers. 

The viscosity of pasta with the addition of β-glucans differed significantly (*p* ≤ 0.05) from that in the control sample (CON) (Table 4). The addition of β-glucans into the products meant that, during the suspension heating process, the maximum viscosity was obtained at 75 °C, while the maximum value of this parameter in the case of the control test was only reached at 95 °C. This relationship may be related to the limited swelling of starch granules and the hampered starch pasting in samples containing β-glucans. A significant decrease in viscosity during suspension heating (75–95 °C) may result from partial depolymerization of β-glucans in the process and a decrease in their molecular weight. A significant (*p* ≤ 0.05) increase in the viscosity of the BG15 and BG20 products was observed after cooling to 50 °C, which seems to be important in terms of the functional properties of enriched pasta. Increased viscosity in the intestine delays glucose and cholesterol absorption and inhibits bile acid reabsorption [54]. The physicochemical properties of β-glucans can affect the digestibility of starch [17,55]. The increase in the viscosity of oat β-glucans caused a decrease in the digestibility of starch [56]. The functionality of β-glucans as conditioned among others by the molecular weight of β-glucan, which needed to be sufficiently high to be capable to increase the viscosity in intestines [54]. The molecular weight of β-glucan was related to acid degradation [57]. To assess the effect of β-glucan-supplemented pasta on intestinal viscosity, the viscosity of the gastrointestinal content should be examined in simulated in vitro digestion studies.

### 3.4. Cooking Quality

Studies conducted by other authors [25,26,27,28,29,30,31] have indicated that the addition of β-glucans reduced the cooking quality of pasta. Our study focused on the impact of adding β-glucans, xanthan gum, and vital gluten simultaneously. The results demonstrated that the amount of β-glucans addition also significantly (*p* ≤ 0.05) affected the cooking quality of pasta samples (Table 5). An increase in the optimal cooking time (OCT) was observed for products enriched with 15% and 20% of β-glucans. The effect of adding soluble fiber (β-glucans/inulin) to extend cooking time has been confirmed by other authors [28,31]. The observed OCT changes may be caused by high water absorption of high-fiber components in products, which compete for water with starch, thereby hindering its swelling and pasting [34,58]. Different results were obtained by Chillo et al. [29], who studied the impact of two commercially available barley β-glucan preparations on the cooking quality of durum wheat spaghetti. They did not notice differences in OCT relative to the control (0% β-glucans) and samples with the variable share of the addition of Glucagel^®^. In contrast, the OCT of the examined samples with the addition of a Bilans ™ preparation increased with the increase in the share of the preparation. In the 10% β-glucans Bilans ™ assay, the OCT was 1.5 min longer than the control. In the present research, the extension of OCT for samples with the addition of β-glucans may have been the cause of the greater losses of dry matter, compared to the control and BG0, as a result of the passage of part of soluble fiber into the solution during cooking [59]. 

It is worth emphasizing that the almost twice lower dry matter loss in the BG0 sample (compared to BG20) may be related to the highest protein content in this sample (Table 2). Protein has a major impact on maintaining a stable product structure and reducing dry matter loss. The protein-starch matrix is associated with maintenance of better integrity during cooking and improvement of the quality parameters of pasta [51]. Increasing dry matter loss accompanying an increase in the proportion of soluble fiber (guar gum/β-glucans) has been observed [26,27]. The weight values for the BG15 and BG20 samples increased significantly (*p* ≤ 0.05) compared to samples with the 0–10% share of β-glucans and the control sample. No statistical differences were observed between the BG0, BG5, and BG10 samples. The increase of the weight of the BG15 and BG20 samples is adequate to the WAI values obtained for these samples (Table 3). Undoubtedly, the ability to absorb water during cooking was related to the extension of the optimal cooking time and the high water capacity of soluble fiber. The reduction of the amount of water available to starch may contribute to its lower pasting capacity and digestibility [26]. In the case of the value increase index, a significant (*p* ≤ 0.05) increase was noted in the BG0 pasta (90% semolina, 5% xanthan gum, and 5% gluten) compared to the control sample. Due to its high value of WAI, xanthan gum contributed to a significant (*p* ≤ 0.05) increase in the volume of the product during cooking. The increase in the β-glucan content (from 0 to 20%) caused a decrease in this parameter.

### 3.5. Color of Pasta

The addition of xanthan gum and vital gluten was responsible for the darker color of uncooked pasta (Table 6). An opposite effect was noted with the addition of β-glucans. The product became brighter (higher values of the L* parameter) as the share of this oat component increased. Pasta samples with xanthan gum and vital gluten additionally enriched with 20% of β-glucans (BG20) were characterized by similar brightness as the control sample (CON) (L = 51.10 and 51.52, respectively, for BG20 and CON). Similar results were obtained by Hajji et al. [60] in their analyses of durum wheat pasta enriched with barley β-glucans (at a level of 1–7%). In the case of the uncooked products, no statistical differences (*p* ≤ 0.05) were observed between the pasta with β-glucans and the control without β-glucans. The addition of xanthan gum and vital gluten negatively affected the intensity of yellowness (b parameter) and redness (a parameter) of the pasta. The fortification of the pasta with β-glucans reduced the negative changes in the color caused by the addition of vital gluten and xanthan gum, but even samples enriched with 20% of beta-glucan were less yellow and less red than the control sample (CON). The analysis of the color of the cooked pasta demonstrated a different effect of the addition of xanthan gum and vital gluten on product brightness (Table 6). Cooked pasta enriched with xanthan gum, vital gluten, and a small addition of β-glucans (5–10%) had a brighter color compared to the control sample (CON). This was probably due to the intense absorption of water by these products during cooking and the significant (*p* ≤ 0.05) increase in the volume index. Consequently, the lower concentration of pigments in the products may have caused the color changes. The increase in the β-glucan addition to the level of 15–20% resulted in a darker color of the cooked pasta; however, it should be emphasized that the values of parameter L for BG15 and BG20 were comparable with those recorded for the control sample (CON). The addition of β-glucans intensify the yellowness of the product; however, pasta samples with up to 20% β-glucans (BG20) were less yellow than the control sample (CON). As reported by Hajji et al. [60], reduction of the intensity of yellowness (b parameter) may be caused by lower protein content in β-glucan compared to that of semolina durum. At the same time, as a function of the increasing proportion of beta-glucans, the volume increase factor declines, which may contribute to a greater concentration of carotenoids in the product - therefore the value of the b parameter and the parameter increases in the case of cooked pasta.

### 3.6. Sensory Quality

The results of the sensory assessment confirmed that even the 20% addition of β-glucans did not deteriorate the sensory quality of the uncooked products (Table 7). All pasta samples with added β-glucans (5–20%) were approved by the study participants. No significant differences were found between uncooked products enriched with β-glucans, BG0, and the control sample (CON). In the case of cooked pasta, there was a risk that the increase in viscosity in samples with the addition of β-glucans would reduce the tastiness of the product [61]. Although there were no significant differences (*p* ≤ 0.05) in the appearance and hardness between the β-glucan-supplemented samples, the BG0 sample, the controls (CON), and the samples supplemented with 20% beta-glucan received significantly lower marks for color, taste, adhesiveness, and springiness, compared to CON and BG0 (Table 7). It should be noted, therefore, that the highest additive of β-glucans that would be acceptable to consumers on a par with the control (CON) is 15% (BG15). Jaworska et al. [62] examined the sensory preferences of consumers of pasta with the addition of oat-β-glucans and reported that pasta with 16% addition of this ingredient turned out to be most often desired among samples with different levels of enrichment (the share of β-glucans was 0, 4, 8, 12, and 16%). The authors revealed that the choice of pasta with the 16% share of β-glucans was determined by the respondents’ interest in the inclusion of dietary fiber in the diet and the need to consume functional products.

## 4. Conclusions

The obtained results have proved that, by using the appropriate additive β-glucans and simultaneously adding vital wheat gluten and xanthan gum, it is possible to obtain a functional product with high quality properties. The inclusion of xanthan gum and vital wheat gluten improved the cooking quality of the pasta. The addition of β-glucans reduced the negative color changes caused by the addition of xanthan gum and vital wheat gluten in the uncooked products. In contrast, the higher levels of β-glucans (15–20%) in the cooked products contributed to the darkening of the pasta and increased the yellowness, but the pasta was still less yellow than the control. The addition of β-glucans significantly increased the viscosity of the pasta. The highest 20% addition of β-glucans did not cause deterioration of the sensory quality of the uncooked products. In the case of the cooked pasta, the large addition of β-glucans (20%) produced a negative increase in adhesiveness and deteriorated the springiness, color, and taste of the product. In summary, the use of a 10–15% additive of β-glucans and 5% additive of vital wheat gluten and xanthan gum yielded functional pasta containing 3.3–5.5 g β-glucans/100g with high cooking quality and sensory attributes.

## Figures and Tables

**Table 1:** The experimental model.

Samples	Raw Materials (%)				Water Addition (g kg^−1^)	Pressure (MPa)	Barrel Temp. (°C)	Extruder Output (kg h^−1^)
	Semolina Durum	Oat β-Glucans	Vital Wheat Gluten	Xanthan Gum				
CON	100	-	-	-	305.2	8.5	28.9	34.68
BG0	90	0	5	5	305.6	13	29.6	29.88
BG5	85	5	5	5	309	13	29.8	31.56
BG10	80	10	5	5	312.4	13	29.8	32.40
BG15	75	15	5	5	315.8	13.5	29.8	32.40
BG20	70	20	5	5	319.4	13.5	29.8	32.38

CON—control sample; BG—β-glucans.

**Table 2:** Chemical composition of raw material and pasta samples.

Samples	Moisture	Protein	Fat	Ash	TDF	IDF	SDF	β-Glucans	Digestible Carbohydrate
	(%)				(g/100 g d.m.)				
Raw materials									
Semolina durum	9.50 ^e^ ± 0.04	13.24 ^c^ ± 0.71	1.1 ^a^ ± 0.04	0.76 ^b^ ± 0.01	3.89 ^abc^ ± 0.15	2.07 ^b^ ± 0.09	1.83 ^ab^ ± 0.06	0.18 ^a^ ± 0.05	81.01 ^e^ ± 1.14
Vital wheat gluten	7.31 ^b^ ± 0.05	70.99 ^g^ ± 3.89	1.48 ^b^ ± 0.03	0.57 ^a^ ± 0.01	23.81 ^e^ ± 0.74	22.69 ^g^ ± 0.96	1.12 ^a^ ± 0.22	nd	3.15 ^b^ ± 0.55
Xanthan gum	11.17 ^h^ ± 0.13	5.97 ^a^ ± 0.38	2.03 ^de^ ± 0.16	8.36 ^g^ ± 0.09	83.36 ^k^ ± 4.9	3.17 ^b^ ± 0.28	80.19 ^f^ ± 1.86	nd	0.28 ^a^ ± 0.25
Oat β-glucans	4.19 ^a^ ± 0.07	8.45 ^b^ ± 0.06	3.56 ^g^ ± 0.08	1.99 ^f^ ± 0.02	33.48 ^f^ ± 1.35	0.64 ^a^ ± 0.2	32.85 ^e^ ± 1.15	27.58 ^f^ ± 0.03	52.52 ^c^ ± 1.51
Pasta samples									
CON	8.83 ^c^ ± 0.16	13.57 ^cd^ ± 0.1	1.67 ^bc^ ± 0.05	1.17 ^c^ ± 0.03	4.45 ^b^ ± 0.27	2.34 ^a^ ± 0.19	2.11 ^b^ ± 0.46	0.19 ^a^ ± 0.07	79.14 ^e^ ± 0.37
BG0	9.05 ^cd^ ± 0.10	17.17 ^f^ ± 0.43	1.84 ^cd^ ± 0.07	1.38 ^d^ ± 0.02	15.16 ^c^ ± 0.27	5.97 ^d^ ± 0.19	9.19 ^c^ ± 0.46	0.16 ^a^ ± 0.01	64.45 ^d^ ± 1.47
BG5	10.33 ^g^ ± 0.06	15.7 ^ef^ ± 0.6	2.23 ^e^ ± 0.08	1.39 ^d^ ± 0.02	15.37 ^c^ ± 0.25	5.41 ^cd^ ± 1.59	9.96 ^cd^ ± 0.26	2.03 ^b^ ± 0.23	65.31 ^d^ ± 2.84
BG10	9.93 ^f^ ± 0.18	15.9 ^ef^ ± 0.78	2.55 ^f^ ± 0.09	1.38 ^d^ ± 0.05	14.87 ^c^ ± 0.39	3.9 ^abc^ ± 0.34	10.97 ^d^ ± 0.04	3.31 ^c^ ± 0.44	65.30 ^d^ ± 1.54
BG15	9.32 ^de^ ± 0.15	15.2 ^de^ ± 0.86	2.7 ^f^ ± 0.09	1.49 ^de^ ± 0.05	15.96 ^cd^ ± 0.12	3.5 ^ab^ ± 0.29	12.47 ^e^ ± 0.17	5.44 ^d^ ± 0.32	64.65 ^d^ ± 1.49
BG20	9.17 ^d^ ± 0.03	15.33 ^de^ ± 0.76	2.74 ^f^ ± 0.09	1.55 ^e^ ± 0.05	18.06 ^d^ ± 0.35	4.23 ^bcd^ ± 0.09	13.83 ^f^ ± 0.44	6.22 ^e^ ± 0.25	62.32 ^d^ ± 1.27

Explanation: d.m.—dry matter; IDF—insoluble dietary fiber; SDF—soluble dietary fiber; TDF—total dietary fiber; nd—not detected; CON—control sample; BG—β-glucans. Data are presented as mean (*n* = 3) ± standard deviation. Data value of each parameter with different superscript letter in the columns are significantly different (Tukey test, *p* ≤ 0.05).

**Table 3:** Physical properties of raw material and pasta samples.

Samples	Equivalent Diameter (µm)	WAI	WSI
		(%)	
Raw materials			
Semolina durum	286.37 ^b^ ± 34	84.7 ^a^ ± 3.7	5.18 ^a^ ± 0.23
Vital wheat gluten	52 ^a^ ± 32	150.3 ^a^ ± 2.9	8.18 ^bc^ ± 0.13
Xanthan gum	25 ^a^ ± 16	2319.3 ^d^ ± 94.7	24.69 ^f^ ± 2.28
Oat β-glucans	83 ^a^ ± 20	900.9 ^c^ ± 17.2	66.26 ^g^ ± 1.9
Pasta samples			
CON	-	100.4 ^a^ ± 2.3	9.5 ^bcd^ ± 0.1
BG0	-	361 ^b^ ± 14.0	7.0 ^ab^ ± 0.1
BG5	-	371.7 ^b^ ± 23.6	9.5 ^bcd^ ± 0.3
BG10	-	376.9 ^b^ ± 4.7	10.9 ^cde^ ± 0.1
BG15	-	377.6 ^b^ ± 4.5	12.2 ^de^ ± 0.1
BG20	-	393.3 ^b^ ± 15.4	12.3 ^e^ ± 0.2

Explanation: WAI—water absorption index; WSI—water solubility index; CON—control sample; BG—β-glucans; Data are presented as mean (*n* = 3) ± standard deviation. Data value of each parameter with different superscript letter in the columns are significantly different (Tukey test, *p* ≤ 0.05).

**Table 4:** The results of the tests of the apparent viscosity of pasta (Pa s) using the shear rate gradient of 1000 s^−1^.

Samples	Heating				Cooling		
	Temp. 65 °C	Temp. 75 °C	Temp. 85 °C	Temp. 95 °C	Temp. 95 °C *	Temp. 50 °C	Temp. 50 °C **
Con	0.006 ^aA^ ± 0	0.007 ^aB^ ± 0	0.010 ^aC^ ± 0	0.012 ^aD^ ± 0.001	0.014 ^aF^ ± 0.001	0.015 ^aG^ ± 0	0.013 ^aE^ ± 0
BG0	0.012 ^bA^ ± 0	0.023 ^bB^ ± 0	0.028 ^bC^ ± 0	0.031 ^cE^ ± 0	0.030 ^cD^ ± 0	0.039 ^bG^ ± 0	0.035 ^bF^ ± 0
BG5	0.013 ^cA^ ± 0	0.034 ^dE^ ± 0.001	0.031 ^cC^ ± 0	0.032 ^dD^ ± 0	0.030 ^cB^ ± 0	0.040 ^cG^ ± 0	0.036 ^cF^ ± 0
BG10	0.014 ^dA^ ± 0	0.029 ^cB^ ± 0	0.032 ^dC^ ± 0.001	0.029 ^bB^ ± 0	0.029 ^bB^ ± 0.001	0.039 ^bE^ ± 0	0.035 ^bD^ ± 0
BG15	0.017 ^eA^ ± 0	0.039 ^eD^ ± 0	0.038 ^eC^ ± 0	0.038 ^fC^ ± 0	0.036 ^dB^ ± 0	0.045 ^eF^ ± 0.001	0.044 ^dE^ ± 0
BG20	0.025 ^fA^ ± 0.001	0.045 ^fE^ ± 0.001	0.041 ^fC^ ± 0.001	0.037 ^eB^ ± 0	0.037 ^eB^ ± 0	0.048 ^dE^ ± 0	0.044 ^dD^ ± 0

Explanation: *—measurement after 20 min, **—measurement after 30 min; CON—control sample; BG—β-glucans; Data are presented as mean ± standard deviation. Data value of each parameter with different uppercase superscript letter in the rows are significantly different (Tuckey test, *p* ≤ 0.05). Data value of each parameter with different lowercase superscript letter in the columns are significantly different (Tukey test, *p* ≤ 0.05).

**Table 5:** Cooking quality of pasta samples.

Pasta Samples	Optimum Cooking Time (min)	Cooking Loss (% d.m.)	Cooking Weight Increase	Cooking Volume Increase
CON	9 ^a^ ± 0.0	4.52 ^c^ ± 0.42	2.54 ^a^ ± 0.04	3.16 ^a^ ± 0.12
BG0	9 ^a^ ± 0.0	2.95 ^a^ ± 0.17	2.73 ^b^ ± 0.02	4.29 ^d^ ± 0.18
BG5	9 ^a^ ± 0.5	3.58 ^b^ ± 0.13	2.77 ^b^ ± 0.04	3.88 ^c^ ± 0.13
BG10	9 ^a^ ± 0.5	3.82 ^b^ ± 0.11	2.74 ^b^ ± 0.02	3.46 ^b^ ± 0.16
BG15	10 ^b^ ± 0.0	5.03 ^d^ ± 0.09	2.88 ^c^ ± 0.01	3.46 ^b^ ± 0
BG20	11.5 ^c^ ± 0.5	5.14 ^d^ ± 0.09	3.09 ^d^ ± 0	3.38 ^b^ ± 0.18

Explanation: d.m—dry matter; CON—control sample; BG—β-glucans; Data are presented as mean ± standard deviation. Data value of each parameter with different superscript letter in the columns are significantly different (Tukey test, *p* ≤ 0.05).

**Table 6:** Color parameters of cooked and uncooked pasta samples.

Pasta Samples	Uncooked			Cooked		
	L *	a *	b *	L *	a *	b *
CON	51.52 ^c^ ± 1.41	2.11 ^d^ ± 0.15	16.22 ^c^ ± 0.96	76.67 ^a^ ± 0.80	−0.43 ^b^ ± 0.11	17.95 ^c^ ± 1.07
BG0	48.44 ^a^ ± 1.90	1.4 ^a^ ± 0.15	14.49 ^b^ ± 1.36	79.14 ^b^ ± 1.42	−0.66 ^a^ ± 0.09	13.93 ^a^ ± 0.61
BG5	49.78 ^ab^ ± 0.94	1.45 ^a^ ± 0.16	13.84 ^ab^ ± 0.56	79.11 ^b^ ± 1.18	−0.73 ^a^ ± 0.10	13.82 ^a^ ± 0.91
BG10	48.98 ^a^ ± 2.13	1.62 ^b^ ± 0.20	13.58 ^a^ ± 0.88	79.11 ^b^ ± 1.40	−0.43 ^b^ ± 0.12	14.52 ^ab^ ± 0.96
BG15	50.24 ^b^ ± 0.73	1.8 ^c^ ± 0.16	13.81 ^ab^ ± 0.84	76.14 ^a^ ± 1.07	−0.30 ^b^ ± 0.09	14.11 ^ab^ ± 0.59
BG20	51.10 ^bc^ ± 0.70	1.84 ^c^ ± 0.10	13.7 ^ab^ ± 0.34	75.95 ^a^ ± 1.59	−0.31 ^b^ ± 0.08	14.92 ^b^ ± 0.92

Explanation: CON—control sample; BG—β-glucans; * - concern CIE-lab color scale. Data are presented as mean ± standard deviation. Data value of each parameter with different superscript letter in the columns are significantly different (Tukey test, *p* ≤ 0.05).

**Table 7:** Organoleptic quality of pasta samples.

Pasta Samples	Uncooked			Cooked						
	Apparance	Color	Odor	Apparance	Color	Taste	Odor	Hardness	Adhesiveness	Springness
CON	4.6 ^a^ ± 0.55	4.2 ^a^ ± 0.45	4.8 ^a^ ± 0.45	3.6 ^a^ ± 0.45	4.2 ^b^ ± 0.45	5 ^b^ ± 0	4.8 ^a^ ± 0.45	4.6 ^a^ ± 0.55	5 ^c^ ± 0	4.8 ^b^ ± 0.45
BG0	4.8 ^a^ ± 0.45	4.4 ^a^ ± 0.45	4.8 ^a^ ± 0.45	5 ^c^ ± 0	4.8 ^c^ ± 0	5 ^b^ ± 0	5 ^a^ ± 0	5 ^a^ ± 0	5 ^c^ ± 0	5 ^b^ ± 0
BG5	5 ^a^ ± 0	4.6 ^a^ ± 0	5 ^a^ ± 0	4.6 ^bc^ ± 0.35	4.6 ^bc^ ± 0.55	4.6 ^ab^ ± 0.55	4.8 ^a^ ± 0.45	4.4 ^a^ ± 0.55	4.8 ^bc^ ± 0.45	4.6 ^b^ ± 0.55
BG10	5 ^a^ ± 0	4.8 ^a^ ± 0.45	4.8 ^a^ ± 0.45	4.6 ^bc^ ± 0.35	4.6 ^bc^ ± 0.55	4.6 ^ab^ ± 0.55	4.8 ^a^ ± 0.45	4.8 ^a^ ± 0.45	5 ^c^ ± 0	4.6 ^b^ ± 0.55
BG15	5 ^a^ ± 0	4.83 ^a^ ± 0.45	4.8 ^a^ ± 0.45	4.0 ^ab^ ± 0.45	4.2 ^b^ ± 0.45	4.2 ^ab^ ± 0.45	4.4 ^a^ ± 0.55	4.8 ^a^ ± 0.45	4.4 ^b^ ± 0.15	4.2 ^ab^ ± 0.45
BG20	4.83 ^a^ ± 0	4.75 ^a^ ± 0.45	4.83 ^a^ ± 0.45	3.8 ^a^ ± 0.35	3.2 ^a^ ± 0.45	4 ^a^ ± 1	4.6 ^a^ ± 0.55	4.4 ^a^ ± 0.55	3.8 ^a^ ± 0.35	3.6 ^a^ ± 1

Explanation: CON—control sample; BG—β-glucans; Data are presented as mean ± standard deviation. Data value of each parameter with different superscript letter in the columns are significantly different (Tukey test, *p* ≤ 0.05).
